# From Caffeine to Aspirin: Everyday Molecules as Triggers for Genetically Encoded Proximity Systems

**DOI:** 10.1002/chem.71530

**Published:** 2026-08-03

**Authors:** Mingguang Cui, Tianlu Wang, Yaoyu Han, Yun Huang, Tien‐Hung Lan, Yunbin Zhou

**Affiliations:** ^1^ Center for Translational Cancer Research Institute of Biosciences and Technology Texas A&M University Houston Texas USA; ^2^ Center for Epigenetics and Disease Prevention Institute of Biosciences and Technology Texas A&M University Houston Texas USA; ^3^ Department of Translational Medical Sciences College of Medicine Texas A&M University Houston Texas USA

**Keywords:** artificial intelligence, chemical induced proximity, immunotherapy, nanobody, over‐the‐counter drugs

## Abstract

Chemically induced proximity (CIP) enables programmable control of gene expression, protein activity, cellular signaling, and engineered cell functions using small molecules. Yet many classical CIP platforms rely on ligands that may limit therapeutic translation owing to unfavorable biosafety profiles, off‐target activity, incomplete reversibility, dosing constraints, and potential immunogenicity of engineered components. This perspective highlights dietary small molecule‐ and OTC drug‐responsive CIP systems as emerging platforms for translational chemogenetic control. We focus on two complementary design strategies. One exploits nanobody‐derived binders against familiar small molecules, as exemplified by synthetic anti‐caffeine nanobody‐based systems. The other adapts naturally evolved ligand‐binding proteins through rational protein engineering, as illustrated by salicylate‐responsive platforms derived from plant salicylic acid (SA)‐binding receptors for reversible proximity control and aspirin‐responsive gene regulation. Together, these engineered systems demonstrate how familiar molecules such as caffeine, SA, and aspirin can be transformed into programmable biological inputs. We further discuss how artificial intelligence (AI)‐guided protein engineering may expand this design space by enabling *de novo* construction and functional reprogramming of ligand‐responsive modules, leading to safer and clinically compatible proximity‐control systems.

## Graphical abstract

This Perspective article highlights how familiar dietary ingredients and over‐the‐counter (OTC) drugs (such as caffeine and its metabolites, SA, and aspirin) can be repurposed as clinically translatable chemical triggers for genetically encoded proximity systems. Through nanobody engineering, AI‐assisted computational protein design, and ligand‐responsive protein engineering, these readily accessible compounds enable programmable and reversible control over ion channels, receptor signaling, synthetic gene circuits, cell fate, CAR‐T cell activity. Together, these advances position common bioactive molecules as powerful molecular inputs for precision therapeutics and next‐generation gene and cell therapies.

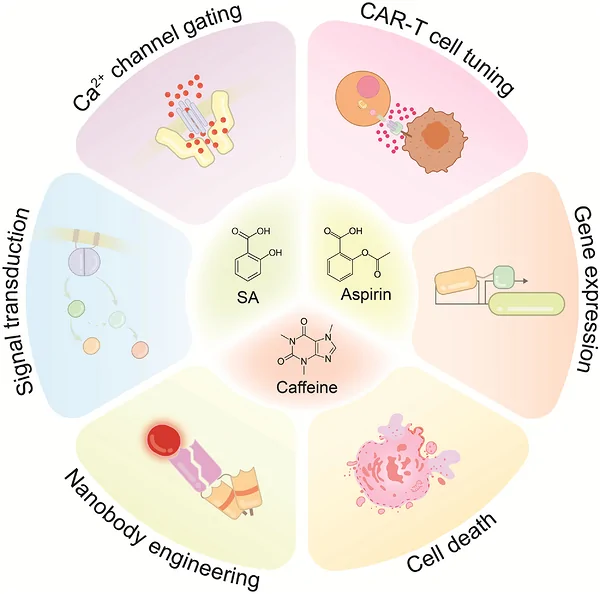

## 1 Introduction

Chemically induced proximity (CIP) systems have become powerful tools for controlling protein function with small molecules [1]. By inducing conditional dimerization, higher‐order assembly, subcellular re‐localization, or split protein reconstitution, CIP technologies enable temporally precise and dose‐dependent control of gene expression, protein activity, cellular signaling, targeted protein degradation, and engineered cell functions [2, 3, 4]. Since the introduction of the rapamycin‐dependent FK506 binding protein (FKBP) / FKBP rapamycin binding domain (FRB) pair [4, 5], the CIP toolbox has expanded to include the abscisic acid (ABA)‐dependent pyrabactin resistance 1‐like (PYL) / ABA‐Insensitive 1 (AB1) system [6, 7], the gibberellin (GA)‐dependent gibberellin‐insensitive dwarf1 (GID1) / gibberellin insensitive (GAI) system [8], the auxin‐dependent transport inhibitor response 1 (TIR1) / transcriptional corepressor auxin/indole‐3‐acetic acid proteins (AID) system [9], calmodulin (CaM) / M13 [10], and other chemically responsive modules [1, 3]. In addition, circular permutation of FRB further produced cpRAPID [11], expanding the rapamycin‐inducible FKBP–FRB platform to chemically controlled transcriptional activation and split‐nanobody reconstitution. More recently, engineered platforms such as INSPIRE and CATCHFIRE have further broadened the design scope of CIP systems by incorporating clinically approved drugs, human‐derived domains, or engineered ligand‐responsive interfaces [12, 13]. Together, these systems have transformed CIP from a set of inducible dimerization tools into a general strategy for programmable control of cellular functions by small molecules.

Despite this progress, translation of CIP technologies into therapeutic settings remains challenging, largely because many established CIP platforms were originally developed as research tools for acute and reversible perturbation rather than as regulatory modules for long‐term or in vivo therapy. In clinical contexts, a useful CIP system must satisfy additional requirements, including inducer biocompatibility, predictable pharmacokinetics, minimal interference with endogenous signaling, low immunogenicity of engineered protein components, and reliable reversibility within a therapeutically relevant dose window [1, 14]. Thus, the limitations of traditional CIP systems arise mainly when they are repurposed for chronic therapeutic control, where ligand pharmacology, protein immunogenicity, dosing flexibility and reversibility, and long‐term safety become as important as switching efficiency (Table 1). For example, rapamycin‐based FKBP‐FRB systems are powerful and highly robust, but rapamycin can perturb endogenous mTOR signaling and immune function [15, 16, 17]. Plant hormone‐based systems such as PYL‐AB1, GID1‐GAI, and TIR1‐AID offer bio‐orthogonality in mammalian cells, yet they rely on exogeneous protein compartments and ligands that were not originally developed for human use and may exhibit unfavorable pharmacological or toxicological properties in mammals [6, 18, 19, 20, 21]. Other chemogenetic systems, including CaM‐M13, INSPIRE, CATCHFIRE, and recently reported nuclear receptor‐based designs, further expand the available chemical inputs, though many still require specialized ligand, extensive protein engineering, or case‐by‐case optimization to balance potency, reversibility, and biocompatibility (Table 1).

**TABLE 1:** Representative CIP systems and their translational potential.

Modules	Chemical inducer	Protein origin	Ligand / PK Profile	Mode of regulation	Translational potential
FKBP‐FRB/cpRAPID	Rapamycin or rapalogs	Human/mammalian‐derived FKBP; engineered FRB	mTOR engagement; immunosuppression [15, 16, 17]	Heterodimerization	Robust and widely validated; limited by endogenous mTOR engagement, immunosuppressive activity, and concerns for long‐term therapeutic use [2, 11, 15, 17]
PYL–ABI1	Abscisic acid	Plant	Nonclinical plant hormone; limited human PK [6, 7]	Heterodimerization	Orthogonal and reversible in mammalian cells; limited by plant‐derived components and lack of clinical validation of ABA as a therapeutic inducer [2, 3, 6, 27]
GID1–GAI	Gibberellin	Plant	Nonclinical plant hormone; limited human PK [8]	Heterodimerization	Orthogonal plant hormone‐based dimerization system; limited by plant‐derived components and nonclinical ligand pharmacology [2, 3, 7, 27]
TIR1–AID	Indole‐3‐acetic acid (IAA)	Plant	Indole‐3‐acetic acid; auxin‐based regulation not optimized for clinical use [9]	Heterodimerization	Powerful for inducible protein degradation; requires ectopic plant‐derived TIR1 and auxin‐based regulation not optimized for clinical use [2, 3, 18, 27]
CaM‐M13	Ca^2^^+^	Human‐derived CaM / peptide	Endogenous Ca^2^^+^ signaling; limited ligand orthogonality [10]	Heterodimerization	Useful for calcium‐responsive regulation; limited by coupling to endogenous Ca^2^^+^/calmodulin signaling and possible perturbation of calcium‐mediated cellular activities [2, 3, 27, 28]
INSPIRE	Clinically approved drugs	Human‐derived ligand‐binding domains	Approved drugs; PK and native target effects are drug‐dependent [13]	Heterodimerization	Uses human‐derived ligand‐binding domains and approved drugs; promising for translational regulation, but may require system‐specific optimization and careful evaluation of drug target effects [13]
CATCHFIRE	Fluorogenic dye	Engineered / nonhuman components	Specialized synthetic dye; limited clinical PK validation [12]	Heterodimerization	Enables high spatiotemporal and fluorogenic control; limited by requirement for specialized synthetic dyes and nonhuman engineered protein components [12]
COSMO/ Caffebody/ CODS	Caffeine/ Theobromine/ Theophylline	Camelid‐derived nanobody‐based	Short caffeine half‐life; CNS stimulant activity; repeated dosing concern [29, 30, 31, 32, 33]	Heterodimerization/ Homodimerization/ Dissociation	Uses a safe and accessible dietary molecule; enables reversible protein dimerization, signaling control, Ca^2^^+^ influx regulation, nanobody reconstitution, CAR‐T cell tuning and cell fate control [2, 29, 30, 31, 32, 33, 34, 35, 36]
Humanized acVHH	Caffeine	Humanized camelid nanobody	Short caffeine half‐life; CNS stimulant activity; repeated dosing concern [29, 30, 31, 32, 33]	Heterodimerization	Combines caffeine responsiveness with heterodimerization and nanobody humanization; improved compatibility for transcriptional control and therapeutic cell engineering [37]
SAMBA	SA or aspirin	Plant‐derived NtNPR1	Salicylate/aspirin exposure; COX and platelet‐related effects [2, 38]	Heterodimerization	Uses clinically familiar salicylates or aspirin and enables reversible proximity control, Ca2+ channel gating, signaling activation, and CAR‐T cell control in vivo; translational use may be limited by the large plant‐derived NtNPR1 fragments and potential immunogenicity [2, 38].
ASPIRIN	SA or aspirin	Plant‐derived AtNPR1/AtNPR4	Aspirin irreversibly inhibits COX/platelet function; bleeding risk with chronic use [39]	Dissociation / Hetero	Aspirin‐responsive gene switch based on AtNPR1/AtNPR4 dissociation; enables transcriptional regulation and therapeutic protein expression in vivo, but relies on plant‐derived protein components whose immunogenicity requires careful evaluation [39].

These considerations point to an important shift in how CIP systems are evaluated for therapeutic use. The performance of a proximity switch is shaped not only by the engineered protein module but also by the pharmacological and practical properties of the chemical inducer itself. For translational applications, factors such as accessibility, cost, pharmacokinetic and pharmacodynamic (PK/PD) behavior, reversibility, off‐target toxicity, and suitability for repeated or long‐term administration become critical design considerations [1, 22]. At the same time, the protein components must be carefully assessed with respect to expression burden, immunogenicity, and compatibility with the intended cellular setting [23, 24]. In practice, ligand selection and protein engineering are therefore closely intertwined, particularly for systems intended for chronic or in vivo therapeutic control [25, 26].

Against this backdrop, dietary small molecules and over‐the‐counter (OTC) drugs have emerged as attractive candidates for translational CIP and chemogenetic applications. These compounds are often inexpensive, orally available, broadly accessible, and supported by extensive human exposure and biosafety profiles. Herein, we highlight the latest advances in dietary small molecule‐ and OTC drug‐responsive CIP systems as representative examples of this emerging direction. Current efforts have largely followed two complementary design strategies. One relies on nanobody‐derived binders against familiar small molecules, exemplified by anti‐caffeine single‐domain nanobody (acVHH)‐based systems that convert caffeine recognition into programmable dimerization, signaling regulation, and gene expression (Table 1). The other adapts naturally evolved ligand‐binding proteins or receptors through splitting, truncation, mutation, and architectural rewiring, as illustrated by salicylate‐responsive systems derived from natural salicylic acid (SA)‐binding receptors in plants. Rather than attempting to exhaustively catalog CIP technologies, we focus on how these approaches illuminate generalizable design principles and how artificial intelligence (AI)‐guided protein engineering may further expand the development of safer and more clinically compatible chemogenetic systems.

### 1.1 Caffeine‐Responsive CIP Systems

Among dietary small molecules, caffeine has emerged as one of the most extensively explored inputs for translationally accessible CIP systems [29]. Widely available from coffee, tea, and other caffeinated beverages, caffeine is inexpensive, commonly accessible, and supported by a well‐established human exposure profile [33, 40, 41]. The molecular basis of caffeine‐responsive CIP systems originates from the anti‐caffeine variable heavy domain of heavy chain (acVHH), which undergoes caffeine‐induced homodimerization with a unique 2:1 binding stoichiometry [42, 43, 44]. This property provides a direct route for converting caffeine recognition into controllable biological outputs and further established caffeine‐responsive nanobodies as a platform for chemogenetic protein regulation [42].

One of the early systems developed from this concept is the caffeine‐stimulated advanced regulators (C‐STAR) system (Figure 1a, left). In C‐STAR, caffeine‐induced acVHH dimerization is coupled to engineered signaling receptors, allowing caffeine to activate intracellular signaling cascades and ultimately induce target gene expression [45]. It is notable that C‐STAR exploits receptor‐mediated signal amplification to translate an upstream ligand‐binder interaction into a robust transcriptional output, rather than directly utilizing caffeine‐induced dimerization as a structural switch. C‐STAR also provided a proof‐of‐concept that a common dietary small molecule could regulate therapeutic protein expression in vivo. In an obesity‐induced type 2 diabetes mouse model, engineered cells controlled by C‐STAR has been utilized to regulate insulin production and improve disease‐associated phenotypes [45]. Despite these strengths, the wild‐type acVHH exhibited only moderate caffeine sensitivity, with an EC50 in the sub‐micromolar range (EC50 = 567.5 nM), motivating subsequent efforts to improve the binder and the overall system architecture.

**FIGURE 1:**
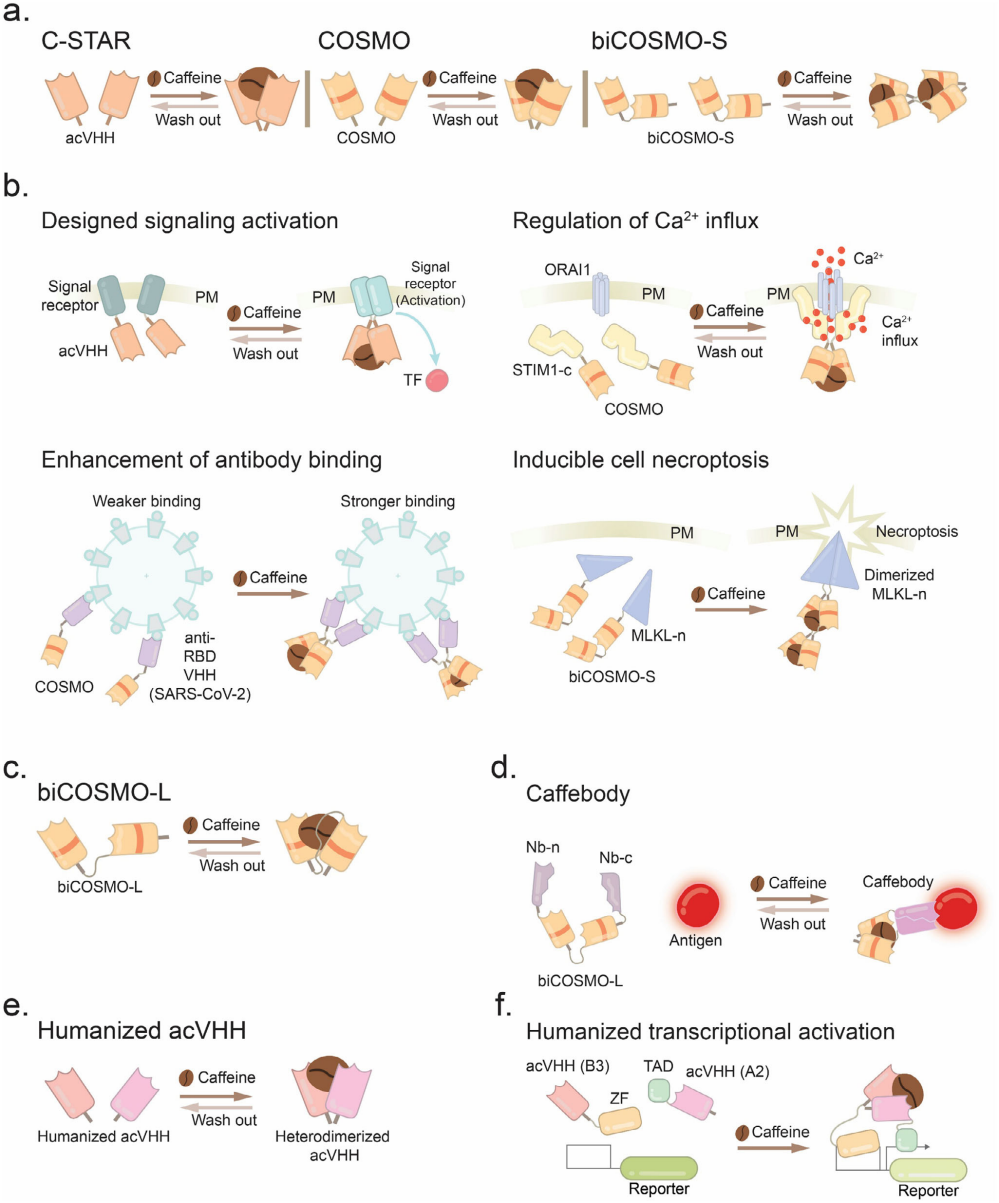
Caffeine‐responsive CIP systems. (a) Caffeine‐induced homodimerization modules based on acVHH. C‐STAR uses the original acVHH scaffold, while COSMO and biCOSMO‐S improved caffeine responsiveness through binding‐interface engineering and bivalent avidity‐enhanced assembly. (b) Representative applications of caffeine‐dependent homodimerization systems. Caffeine‐induced proximity has been used to activate engineered signaling receptors, regulate calcium influx through COSMO‐integrated STIM1‐ORAI coupling, enhanced nanobody binding for SARS‐COV‐2 spike protein neutralization, and induced necroptotic cell death. (c) biCOSMO‐L is generated by connecting two COSMO molecules with a long linker, which favors caffeine‐induced intramolecular control. (d) The intramolecular switching effect of biCOSMO‐L enables allosteric protein function control, as illustrated by caffeine‐dependent reassembly of split nanobody fragments for antigen recognition (caffebody). (e) Humanized acVHH systems combine acVHH heterodimerization engineering with nanobody humanization, converting the original acVHH into more clinically compatible caffeine‐responsive modules with reduced homodimerization. (f) Humanized and heterodimeric acVHH pairs can be combined with human‐derived transcriptional components to achieve caffeine‐dependent TF activation. acVHH: anti‐caffeine single‐domain nanobody; PM: plasma membrane; TF: transcription factor; C‐STAR: caffeine‐stimulated advanced regulators (C‐STAR); COSMO: caffeine‐operated synthetic module; biCOSMO‐S: bivalent COSMO with a short linker; biCOSMO‐L: bivalent COSMO with a long linker; STIM1‐c: C‐terminus of stromal interaction molecule 1; RBD: recognize the receptor‐binding domain; MLKL‐n: N‐terminus of mixed lineage kinase domain‐like pseudokinase; Nb: nanobody; ZF: zinc finger; TAD: transactivator domain.

Structure‐guided engineering of acVHH led to the caffeine‐operated synthetic module (COSMO), generated by introducing the Y104W mutation into acVHH via systematic mutational studies on the caffeine‐binding pocket (Figure 1a, middle). COSMO exhibits improved caffeine responsiveness (EC50 = 95.1 ± 1.2 nM) while preserving reversible homodimerization behavior [34]. Further engineering produced bivalent COSMO designs, including biCOSMO‐S and biCOSMO‐L. In biCOSMO‐S, two COSMO modules are connected by a short linker to enhance intermolecular association through avidity effects (Figure 1a, right), resulting in highly sensitive caffeine‐dependent association between individual biCOSMO‐S (EC50 = 16.9 ± 2.0 nM). Together, COSMO and biCOSMO‐S show that caffeine‐responsive CIP performance can be tuned at two levels: affinity optimization of the individual ligand‐binding module and valency control of the larger protein architecture. The applications of COSMO and biCOSMO‐S extend beyond simple dimerization. As summarized in Figure 1b, caffeine‐induced COSMO association has been used to activate engineered signaling pathways, modulate extracellular calcium influx through endogenous ORAI calcium channels [46, 47, 48, 49, 50, 51, 52, 53], induce necroptosis through dimerization of the N‐terminal effector domain of mixed lineage kinase domain‐like pseudokinase (MLKL) [54, 55, 56], and enhance nanobody avidity to more potently neutralize the SARS‐COV‐2 spike protein [34].

While biCOSMO‐S enhances intermolecular association, biCOSMO‐L uses a longer linker to favor caffeine‐dependent intramolecular dimerization [34, 35] (Figure 1c). This design shifts the function of the module from classical intermolecular association toward allosteric control within a single polypeptide. Through inserting biCOSMO‐L into a nanobody, caffebody has been engineered to confer caffeine sensitivity to multiple nanobody scaffolds [35] (Figure 1d). This strategy is conceptually important because it expands caffeine‐responsive homodimerization to reversible allosteric control of protein‐target heterodimerization.

The original acVHH‐based homodimerization platforms nevertheless have limitations that might hinder broader therapeutic applications. Homodimerization is generally suffered from elevated basal activity, undesired self‐assembly, or incompatible with complicate systems that require asymmetric complex formation. In addition, the wild‐type acVHH modules are camelid‐derived, raising potential immunogenicity concerns when expressed in therapeutic cells. To address these issues, humanized and heterodimeric caffeine‐responsive nanobody pairs have been developed (Figure 1e). Heterodimerization favors productive asymmetric assembly and reduces unwanted homodimeric interactions, while humanization decreases the number of nonhuman residues outside the complementarity‐determining regions [37]. These modules were combined with human‐derived signaling components to control transcription factor activation (Figure 1f). In T cells, this system enabled caffeine‐inducible expression of chimeric antigen receptors (CAR) at caffeine concentrations compatible with daily coffee consumption. Thus, the key advance of this work is not only the development of heterodimeric acVHH, but also the shift toward more human‐compatible architectures for therapeutic cell engineering.

Taken together, caffeine‐responsive systems illustrate a clear progression from ligand‐induced nanobody dimerization to increasingly sophisticated chemogenetic control modules. C‐STAR established caffeine‐triggered acVHH homodimerization as a reversible input for signaling and gene regulation, whereas COSMO and biCOSMO‐S improved sensitivity through affinity and valency engineering. biCOSMO‐L further expanded its application from intermolecular association to intramolecular allosteric control, and humanized heterodimeric variants have begun to address potential immunogenicity of the original camelid‐derived homodimeric acVHH scaffold. Collectively, these advances define an emerging design logic in which binder discovery, affinity tuning, multivalency, allosteric switching, heterodimerization, and humanization are integrated to convert a familiar dietary molecule into a versatile therapeutic chemogenetic input for diverse biological functions, including gene expression, calcium influx, and CAR‐T cell regulation. Although these applications differ in downstream outputs, they are enabled by shared chemical principles, including ligand‐dependent complex formation, cooperativity, and proximity‐driven activity, that also underpin other induced‐proximity modalities such as targeted protein degradation [22, 57].

### 1.2 Salicylate‐Responsive Systems

In parallel with caffeine, salicylate‐based compounds represent another attractive class of clinically familiar chemical inputs. SA and acetylsalicylic acid, better known as aspirin, are inexpensive and widely used compounds with long clinical histories and well‐characterized pharmacological profiles [30, 31, 32]. Unlike caffeine‐responsive systems, which originated from nanobody‐derived binder discovery, salicylate‐responsive systems were developed by engineering naturally evolved ligand‐binding proteins or receptors in plants [58, 59, 60]. This distinct design strategy represents a second route to the development of OTC drug‐responsive CIP systems (Table 1).

A notable example of this strategy is the development of the SA‐mediated binary association (SAMBA) system (Figure 2a). SAMBA repurposes tobacco nonexpressor of pathogenesis‐related genes 1 (NtNPR1), a plant SA receptor, into a reversible heterodimerization platform by splitting its SA‐binding core into two complementary fragments [38]. In the absence of ligand, these fragments remain separated, whereas SA addition induces fragments reassembly, thereby realizing ligand‐dependent proximity. This strategy differs fundamentally from caffeine‐responsive acVHH systems, which rely on ligand‐induced nanobody homodimerization. SAMBA therefore broadens the conceptual design space for translational CIP by showing that a natural receptor can be converted into a synthetic proximity switch through structure‐guided engineering.

**FIGURE 2:**
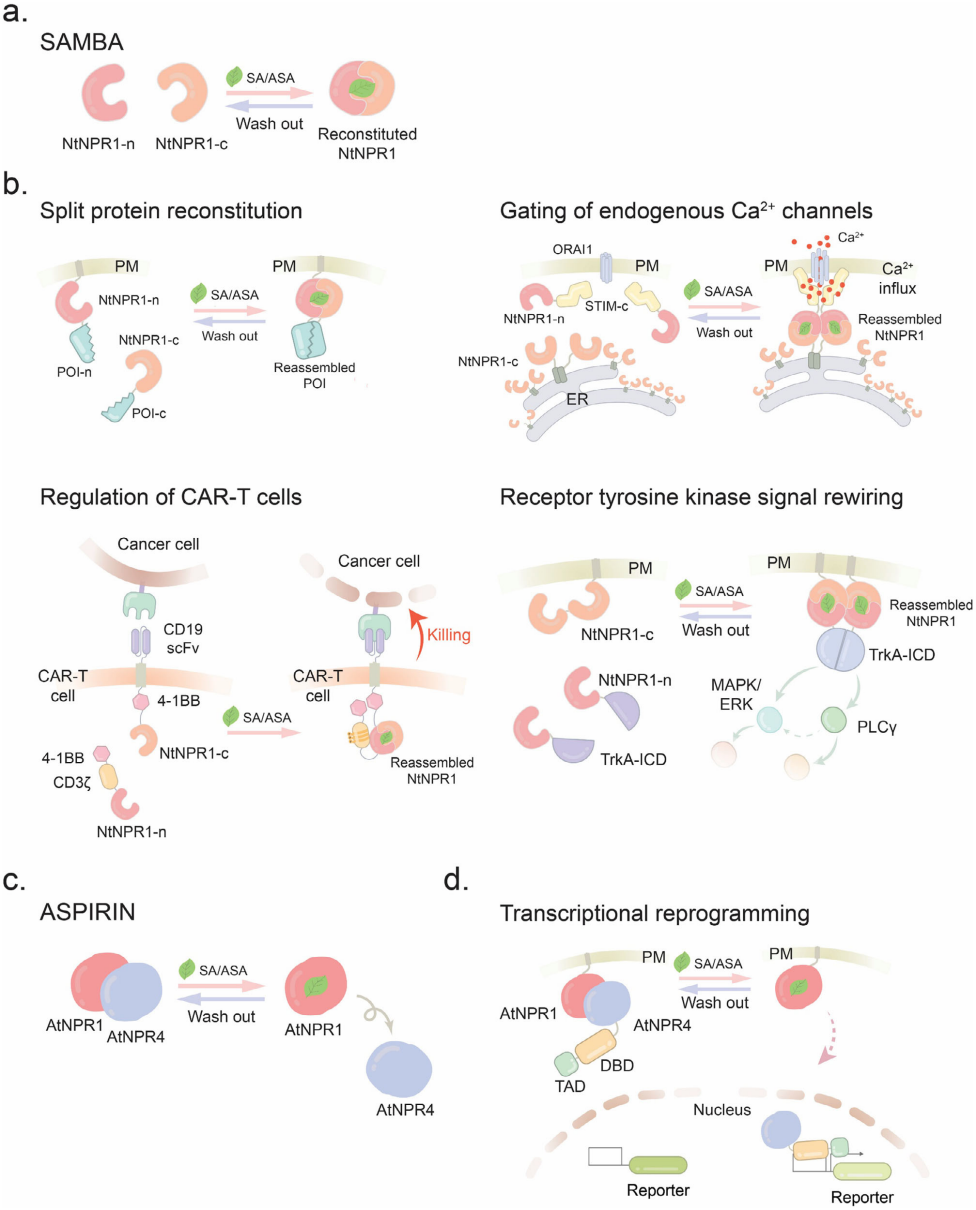
Salicylate‐ or aspirin‐responsive CIP systems. (a) SAMBA is a salicylic acid (SA)‐ or aspirin (ASA)‐dependent binary association system generated by splitting the SA‐binding core of NtNPR1 into two complementary fragments. In the presence of SA, the N‐ and C‐domains of NtNPR1 reassemble to form a heterodimerization module. (b) Representative applications of SAMBA. SA‐induced NtNPR1 fragments reassembly enables split‐protein reconstitution, regulation of graded Ca^2^^+^ influx through STIM1‐ORAI coupling, designed signaling activation through dimerization of the intracellular kinase domain of TrkA‐ICD and tunable CAR‐T cell activation. (c) The ASPIRIN system uses the AtNPR1 and AtNPR4 to build an aspirin‐responsive gene switch. In contrast to SAMBA, which induces association of separated NtNPR1 fragments, ASPIRIN operate through ligand‐induced dissociation of a preassembled AtNPR1‐AtNPR4 complex. (d) In the ASPIRIN transcriptional control system, AtNPR1 anchors AtNPR4 fused to a synthetic transcription factor toward the PM in the absence of aspirin. Then aspirin disrupts the AtNPR1 and AtNPR4 interaction, hence allowing the AtNPR4 linked transcription factor translocating to the nucleus and activating reporter gene expression. SA: salicylic acid; PM: plasma membrane; SAMBA: SA‐mediated binary association; ASPIRIN: advanced free synthetic platform inducible by risk input medication POI: protein of interest; NtNPR1: tobacco derived nonexpressor of pathogenesis‐related genes 1; AtNPR1 and AtNPR4: Arabidopsis thaliana derived nonexpressor of pathogenesis‐related genes 1 and 4; STIM1‐c: C‐terminus of stromal interaction molecule 1; 4‐1BB: intracellular domain of tumor necrosis factor receptor superfamily member 9 (TNFRSF9); TrkA‐ICD: intracellular domain of tropomyosin receptor kinase A; MAPK/ERK: mitogen‐activated protein kinase/ extracellular‐signal‐regulated kinase; PLCγ: phospholipase C gamma; DBD: DNA binding domain; TAD: transactivator domain.

The development of SAMBA also illustrates why translational CIP systems should be optimized for realistic exposure windows rather than maximal affinity alone. Through iterative rational mutagenesis and structural optimization, SAMBA achieved substantially improved sensitivity to SA while maintaining responsiveness within clinically relevant concentration ranges (0.05‐0.5 mM) [38]. The system was tuned to avoid undesired activation by low background salicylate levels that could arise from dietary exposure, while remaining responsive to pharmacologically meaningful SA concentrations. This balance is essential for any system intended for in vivo use, where background ligand exposure and patient variability may influence basal activity.

One of the most attractive features of SAMBA is its rapid reversibility, with ligand washout immediately promoting fragments dissociation [38]. This dynamic behavior enabled temporal control across several applications, such as split‐protein reconstruction, STIM1/ORAI1‐based regulation of calcium influx, SA‐gated activation of receptor kinase receptors, and conditional control of chimeric antigen receptor T (CAR‐T) cell activity (Figure 2b). The SAMBA‐CAR T cell design strongly supports the translational potential of salicylate‐responsive proximity systems, as SA‐gated receptor assembly enabled controllable tumor‐cell killing while mitigating cytokine release syndrome, a side effect often seen in patients receiving conventional CAR‐T cell therapy [38]. In general, SAMBA highlights three important principles for translational switch design: First, natural ligand‐binding domains can be repurposed into synthetic CIP modules through structural‐guided engineering. Second, therapeutic systems must be tuned to a clinically useful dose range rather than simply to the strongest possible binding affinity. Third, fast reversibility is valuable when precise temporal control and safety are priorities.

The advanced free synthetic platform inducible by risk input medication (ASPIRIN) system extends this salicylate‐responsive design strategy from proximity control to transcriptional gene regulation (Figure 2c). In contrast to SAMBA, which uses SA to promote reassembly of split NtNPR1‐derived fragments, ASPIRIN harnesses SA‐ or aspirin‐induced dissociation of the Arabidopsis NPR1/NPR4 complex, composed of AtNPR1 and AtNPR4 [39]. In the ASPIRIN design, membrane‐anchored AtNPR1 sequesters an AtNPR4‐fused synthetic transcription factor at the plasma membrane in the absence of ligand, whereas ligand addition disrupts the AtNPR1/AtNPR4 interaction, enabling nuclear translocation of the AtNPR4‐fused transcription factor and subsequent activation of target gene expression. (Figure 2d). This release‐gated mechanism broadens the conceptual scope of CIP‐inspired regulation by showing that chemical control can be achieved not only through induced association, but also through programmed dissociation. Importantly, the demonstrated use of ASPIRIN to regulate insulin expression in diabetic contexts illustrates how salicylate inputs can be connected to therapeutically relevant gene‐control outputs [39]. Together with SAMBA, it shows how plant‐originated ligand‐binding proteins can be repurposed into programmable control systems that operate through either assembly‐ or release‐based mechanism in mammals.

### 1.3 Translational Considerations: Ligand Pharmacology and Protein Immunogenicity

The broad availability, ease of oral administration, low cost, and long history of human use associated with dietary small molecules and OTC drugs make them attractive inputs for clinically oriented CIP systems. Nevertheless, clinical familiarity should not be equated with pharmacological neutrality. When such compounds are repurposed as repeated or long‐term triggers for engineered cellular programs, their native target engagement, pharmacokinetics, dosing frequency, background exposure, and patient‐to‐patient variability in metabolism may all constrain the operational therapeutic window [29, 30, 31, 32, 33].

Caffeine exemplifies this trade‐off. Although it is orally administered and generally well tolerated at customary intake levels, its intrinsic stimulant activity, relatively short systemic persistence, and variable metabolism among different individuals may complicate applications that require sustained or highly standardized activation, particularly when repeated dosing is needed to maintain circuit output [29, 61]. Habitual consumption may further blur the distinction between background exposure and intentional dosing. Conversely, rapid clearance of caffeine and reversibility of caffeine‐responsive modules may be advantageous for transient activation, intermittent treatment, or safety‐switch applications [33]. Accordingly, caffeine‐responsive modules should be calibrated to minimize unintended activation by routine consumption while preserving robust responses within clinically acceptable exposure ranges. Salicylate‐ and aspirin‐responsive systems present a distinct set of constraints. Aspirin irreversibly inhibits cyclooxygenases activity and suppresses platelet function; consequently, repeated or prolonged administration may be unsuitable for patients with increased risk of gastrointestinal injury or bleeding [30, 31, 62]. Taken together, clinical translation may therefore require application‐specific tuning of switch sensitivity, intermittent dosing regimens, or engineered analogs that retain proximity‐inducing activity while minimizing native pharmacological effects.

The immunogenicity of the genetically encoded components represents a separate translational challenge. The original caffeine‐responsive modules are derived from camelid acVHH scaffolds, whereas SAMBA and ASPIRIN incorporate tobacco‐ or Arabidopsis‐derived NPR proteins. Although these nonhuman components provide valuable orthogonality in mammalian cells, their expression may promote immune recognition, particularly in long‐lived therapeutic cells or direct in vivo applications designed to persist for months or years [23, 26]. Humanization of acVHH illustrates one route for mitigating this risk, but comparable strategies remain to be established for plant‐derived salicylate receptors. Potential approaches include epitope deimmunization, minimization of nonhuman sequences, transient or tissue‐restricted expression, and reconstruction of ligand‐responsive interfaces on human‐compatible scaffolds [26, 37, 63]. Collectively, ligand pharmacology and component immunogenicity define key translational constraints that must be addressed in the development of clinically deployable CIP systems.

### 1.4 Future Directions: Toward AI‐Guided Design of Translational CIP Systems

The systems discussed above suggest two complementary paths for engineering translational CIP platforms: nanobody‐based recognition of familiar small molecules and engineering of natural ligand‐binding proteins into synthetic regulatory modules. Future progress will depend not only on identifying clinically compatible ligands but also on developing scalable and modular approaches to generate and optimize ligand‐responsive modules [30, 64]. Traditional approaches based on iterative mutagenesis and experimental screening remain powerful, but they can be labor‐intensive and difficult to generalize across diverse small molecules [65].

Computational design frameworks such as AlphaProteo [66], BindCraft [67], BoltzGen [68], Latent‐X [69], and related platforms illustrate the growing ability to design or optimize protein interfaces against diverse molecular targets [63, 70, 71, 72]. For translational CIP, however, the design problem is broader than creating a high‐affinity binder. Useful systems must also exhibit appropriate ligand sensitivity, low basal activity, tunable cooperativity, reversible kinetics, minimal off‐target assembly, low immunogenicity, and compatibility with clinically achievable ligand concentrations [73]. AI‐guided design will therefore be most valuable when it is integrated with experimental screening and system‐level optimization rather than used as a stand‐alone substitute for validation.

Early examples already illustrate how AI‐guided protein design can expand the CIP toolkit. In one recent macrocycle‐induced dimerization platform, a synthetic macrocyclic ligand served as the design input for generating a cognate protein homodimer that assembles only in the presence of the macrocycle [74]. This work implies a ligand‐first route to CIP development, in which a defined synthetic molecule is used to guide the creation of a new protein‐protein interface capable of controlling gene expression. In parallel, AI‐designed binders can also reprogram existing ligand‐responsive modules into new modes of chemical control. A recent example is the caffeine‐operated dissociation system (CODS), which uses AI‐designed binders to rewire COSMO from a caffeine‐induced dimerization module into a dissociation system []. In CODS, the designed binder associates with COSMO in the absence of caffeine, whereas caffeine‐induced COSMO dimerization competitively disrupts this interaction, resulting in rapid and reversible protein dissociation. Functionally, CODS enabled caffeine‐dependent control of gene expression, induction of programmed pyroptotic cell death, and deactivation of CAR‐T cell activity as a safety OFF‐switch [36]. Together, these examples suggest that AI‐guided design may support next‐generation CIP engineering, through either de novo construction of ligand‐induced dimerizers around defined chemical inputs, or functional reprogramming of existing ligand‐responsive modules into new modes of chemical control.

Moving forward, the field would benefit from evaluating new systems against a shared translational framework. Key criteria should include ligand availability and cost, biosafety profiles, clinically achievable dose range, compatibility with repeated dosing, immunogenicity of the genetically‐encoded components, and robustness in disease‐relevant contexts [14, 75]. Established CIP platforms remain indispensable research tools and performance benchmarks, whereas dietary and OTC inputs offer a complementary route toward practical clinical control when their pharmacology matches the intended use [76]. In summary, these advances position next‐generation CIP systems to convert everyday compounds and food‐derived molecules into clinically deployable control signals for programmable biology, enabling precise, reversible, and scalable regulation of gene and cell therapies.

## Conflicts of Interest

The authors declare no conflicts of interests.
